# Robot-assisted laparoscopic descending colon carcinoma resection with D3 lymph node dissection using the triple bipolar technique and intracorporeal delta anastomosis

**DOI:** 10.1007/s10151-024-02995-3

**Published:** 2024-09-11

**Authors:** J. Mazaki, T. Ishizaki, Y. Kuboyama, R. Udo, T. Tago, K. Kasahara, K. Iwasaki, Y. Nagakawa

**Affiliations:** Department of Gastrointestinal and Pediatric Surgery, Tokyo Medical University, 6-7-1 Nishishinjuku, Shinjuku-Ku, Tokyo, 160-0023 Japan

Although robot-assisted laparoscopic surgeries (RALS) for colonic cancer resection are on the rise, descending colon cancer is the least common type of colorectal cancer and the surgical techniques have not yet been standardized. Unique features of descending colon cancer resection include: (i) lymph node dissection needs to be performed, including the lymph nodes in the area surrounding the root of the inferior mesenteric artery (defined as station 253 lymph node in Japanese Society for Cancer of the Colon and Rectum (JSCCR) guidelines [1], and completing a medial approach while preserving the inferior mesenteric artery (IMA); (ii) the potential need for mobilization of the splenic flexure mobilization and/or rectum depending on the location of the tumor, with a wide range of dissection required. Considering these characteristics and the advantages of RALS, we have standardized a procedure using a triple bipolar technique and intracorporeal delta anastomosis (IA).

We provide a video demonstration of our standardized technique, which involves docking from the right side of the patient, using a Pfannenstiel incision and a 5-port configuration with a 12-mm auxiliary port in the left mid-abdomen. The patient is positioned with a head-down/right-down tilt of 10–15°. The technique involves the following steps: (i) Dissection and mobilization of the mesentery of the descending colon. (ii) Dissection and mobilization of the mesentery of the sigmoid colon. (iii) Lymph node dissection including station 253 lymph nodes (dissection of the main feeder). (iv) Completion of a medial approach with a wide field of view. (v) Lateral approach. (vi) Mesenteric resection and IA (delta anastomosis). Since a vessel sealer is already present on the 4th arm, dissection of the inferior mesenteric vein (IMV) and mesentery can be performed on the resection side without the need to change the forceps. The operating time was 215 min, the console time was 183 min, and blood loss was 15 ml.

Using IA allows surgery with minimal dissection and the possibility of avoiding mobilization of the splenic flexure or rectum.

## Supplementary Information

Below is the link to the electronic supplementary material.

Supplementary file1

## Data Availability

The data that support the findings of this study are available from the corresponding author, J.M., upon reasonable request.
